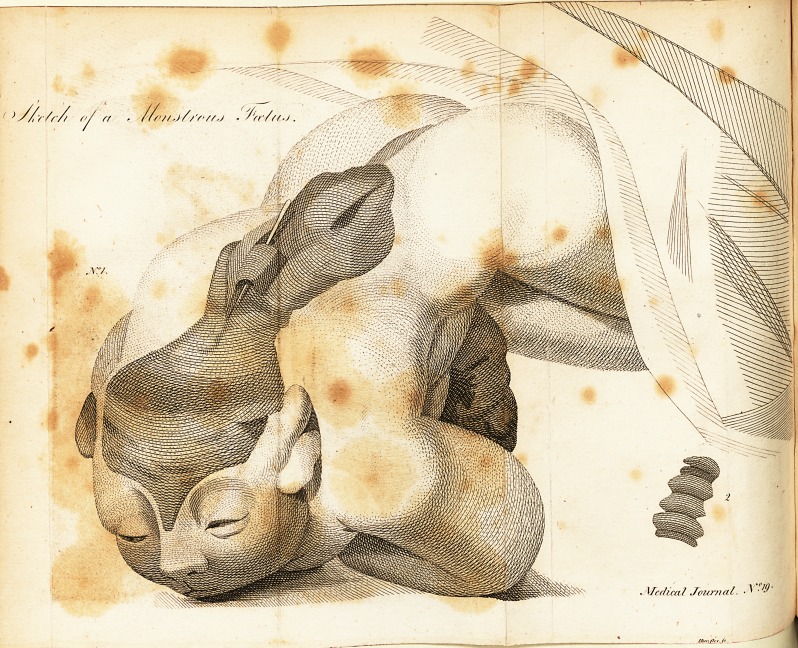# A Case of Mal-Formation

**Published:** 1800-09

**Authors:** W. Simmons

**Affiliations:** Manchester


Medical and Phyfical Journal.

VOL. IV.] SEPTEMBER, 1800. [NO. XIX.

### *A Case of Mal-formation, with an Engraving.*

To the EDITORS of the MEDICAL and PHYSICAL JOURNAL.

Gentlemen,

THAT peculiarity in the fœtus, which confifts in its deprivation of brain, is not very uncommon; I met with an inftance of it eight years ago, and have feen others in different anatomical collections. The deficiency in the fubject I am about to defcribe, is extended to the medulla fpinalis; and as it is new to me, and has given rife to fome reflections, which may not be altogether ufelefs, I have taken the liberty to tranfmit them for infertion in your Journal.

The mother was delivered at Bolton, by Mr. Barlow, to whom I am indebted for the hiftory of the cafe, and for the fœtus itfelf. The only material circumftance in the former, was, the woman's pofitive affertion that fhe went two months over her time, which, however, I am inclined to doubt, and would rather fuppofe, that fhe had committed an error in her reckoning. In the cafes moft analogous, the birth has been ufually premature, generally at the feventh month, and it happened fo in my former cafe.

In this child, the upper part of the cranium is entirely wanting; and there remains only a thin plate of bone, covered with a doubling of membrane, in place of the cervical and the greater part of the dorfal vertebræ. This fold contained no medulla, though it exhibited, on being flit open, fome flender fibrils, which might be conftrued into nerves; I fhould compare it to the proper coverings of the medulla fpinalis, of a thinner texture. Lower down, a difplaced portion of vertebræ is fhewn, which was hollow, but contained no medulla; the reft of the fpine confifted of a folid column of bone, without any fpinous proceffes. The child had, befides, a flight inverfion of the feet, and a hare-lip on the right fide; in other refpects, it was full grown, and the colour of the fkin was natural.

There did not appear to be any deviation from the common ftructure and arrangement in the vifcera of the thorax and abdomen: the heart, lungs, and thymus, occupying the former cavity, in their proper order; and the ftomach, liver, fpleen, kidnies, great and fmall inteftines, &c. the latter. The larger inteftines were alfo diftended with meconium.

In the cheft too, I traced the phrenic nerves, defcending to the diaphragm in their ufual courfe; and in the neck, the par vagum, with its ganglia and intercoftal, lying between the carotid artery and internal jugular vein. Though the eyes were outwardly well formed, I could not find by diffection any optic nerve.

The nerves in the upper and lower extremities were, neverthelefs, perfect; for I traced them in the arm and in the thigh, and in neither did I obferve any difference in their number, fize, colour, or diftribution.

This fœtus was ftill born, which, if I miftake not, has always been the cafe when the brain has been wanting. However, the mother was not fenfible, during pregnancy, of any difference from what fhe had been formerly accuftomed to, either in her own feelings or in the motion of the child; and fhe had had many children. The birth was marked by no particular occurrence; it would probably be facilitated by the reduced bulk of the head.

In comparing the defective ftructure of this child with the afcertained ufes in others of thofe parts of which it was deprived, I have been led to conclude, that nervous influence is not at all neceffary to the growth of the fœtus in utero. At an early period after conception, it is highly probable, that the augmentation of the fœtus is maintained by the circulating fyftem alone; and as it is felf-evident from this cafe, that it can go on at a later, without either brain or fpinal marrow, the nerves muft grow like the other component parts of the body, and perfectly diftinct from any other influence than that of the circulation.

It is proved by experiment, that when the fpinal marrow, or principal nerves of a limb are divided, the parts below are immediately deprived of their fenfibility, and become torpid; hence, we may reafonably infer, that no peculiar property is refident in the nerves themfelves. Affuming then, that the nerves ferve merely to convey the influence of the brain and medulla fpinalis, it is obvious, that when deprived of thefe fources, they can impart none. Thus, it is evident, that although this fœtus had attained the full fize, and its motions were not perceptibly different from another, yet, having no fenforium, it could poffefs no fenfation.

Throughout

Throughout all Nature we obferve the wifdom of Providence, in adapting the ftructure of every animal to its peculiar mode of exiftence. In the fœtus, we note feveral contrivances for the uterine ftate, which become unneceffary foon after birth; as the foramen ovale, the funis umbilicalis, and the ductus arteriofus; the thymus too may be numbered, though its ufe be at prefent unknown: others, as the lungs, then lie dormant, and are called into activity by its change of condition. But, to beftow their proper functions on the nerves, would then be at leaft a work of fupererogation, as there is no object to which the impulfe derived from fenfation could be deftined. On the contrary, fenfibility would expofe it to hazards, which Nature has been fedulous to avert, by depriving the funis of nerves, and by furrounding it with the liquor amnii.

Mr. Barlow informs me, that he has repeatedly tried the experiment upon a prefenting upper or lower extremity, and that the refult has always confirmed my opinion.

As far as I have been able to determine, fenfation is coeval with refpiration; for when, after birth, refpiration has been delayed, and during the pulfation in the funis no appearance of fenfation has arifen till the child began to breathe, the functions of the lungs, and of the nervous fyftem, were then roufed into activity in the fame moment of time.

I purpofely avoid any further difcuffion, though the fubject is pregnant with much curious matter. My object now has been to prove,

1. That nervous influence is not at all neceffary to the growth of the fœtus in utero; and,

2. That the fœtus in its uterine ftate does not poffefs fenfation.

The figures will require but little explanation. In No. 1, the dark colour, as it appeared in the fubject, in the direction of the brain and fpine, fhews the deficiences in thofe parts; and the probe, the hollow portion of vertebræ. A little lower down, the light reprefents the protuberance of the fpine, the folid ftructure of which is delineated in figure, No. 2; and under the left arm, is given a portion of inteftine, diftended with meconium. A cloth was thrown over the legs, becaufe it was thought unneceffary to reprefent them. The natural and healthy appearance of the fkin is preferved, and the maturity of growth well difplayed. The nerves have not been delineated, as they contained nothing remarkable, either in their ftructure or diftribution.

*Manchefter, July* 29, 1800. W. SIMMONS.

N. B. The Drawing is made of the natural fize.

## Figures and Tables

**No. 1. 2 f1:**